# Supplementary material for: Conversion of the bronchial tree into a conforming electrode to ablate the lung nodule in a porcine model
Source: Commun Med (Lond). 2023 Sep 29;3:129. doi: 10.1038/s43856-023-00362-1 (PMC10541426; doi:10.1038/s43856-023-00362-1)
Supplement: Supplementary file 2 — Supplemental information [file 43856_2023_362_MOESM2_ESM.pdf]

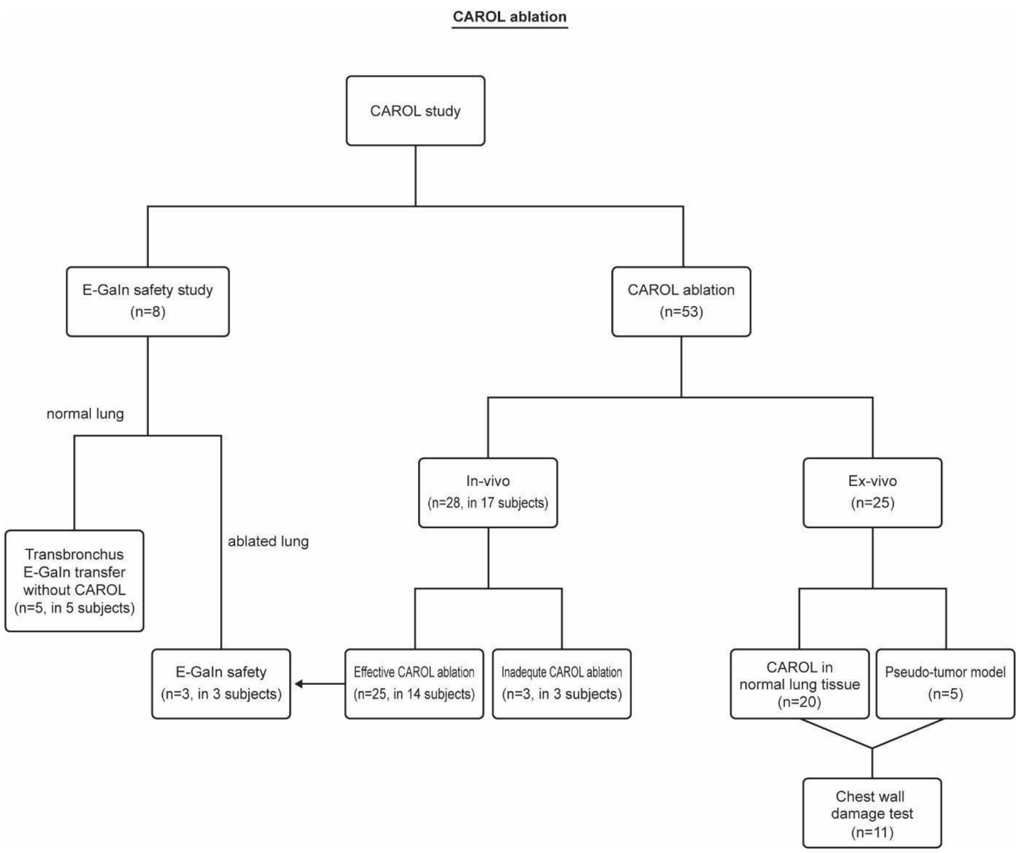

Fig. S1. Experimental schematic diagram of CAROL ablation.

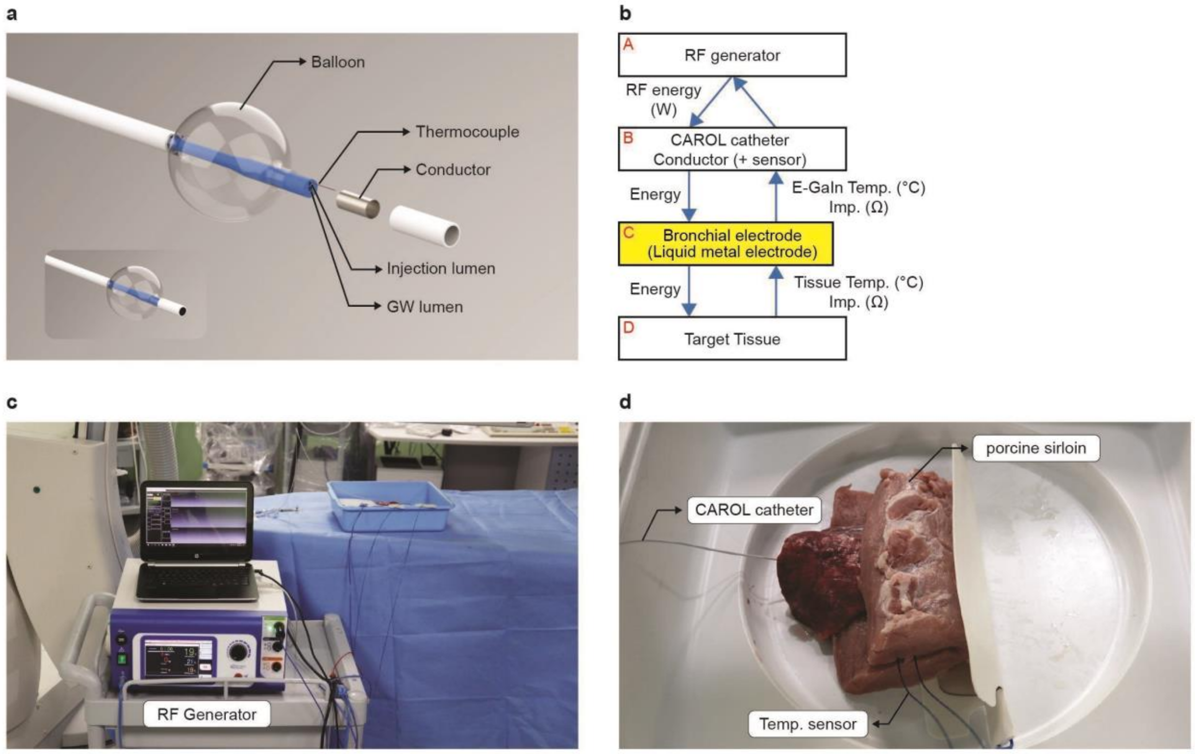

Fig. S2. Composition of the CAROL catheter and ex-vivo experimental method (**a**) CAROL catheter. (**b**) The working process of CAROL ablation under temperature controlled mode (**c**) The ex vivo porcine lung experiment performed under fluoroscopic imaging guidance. (**d**) Experimental setting of the ex vivo porcine lung experiment with porcine sirloin muscle flap mimicking the chest wall.

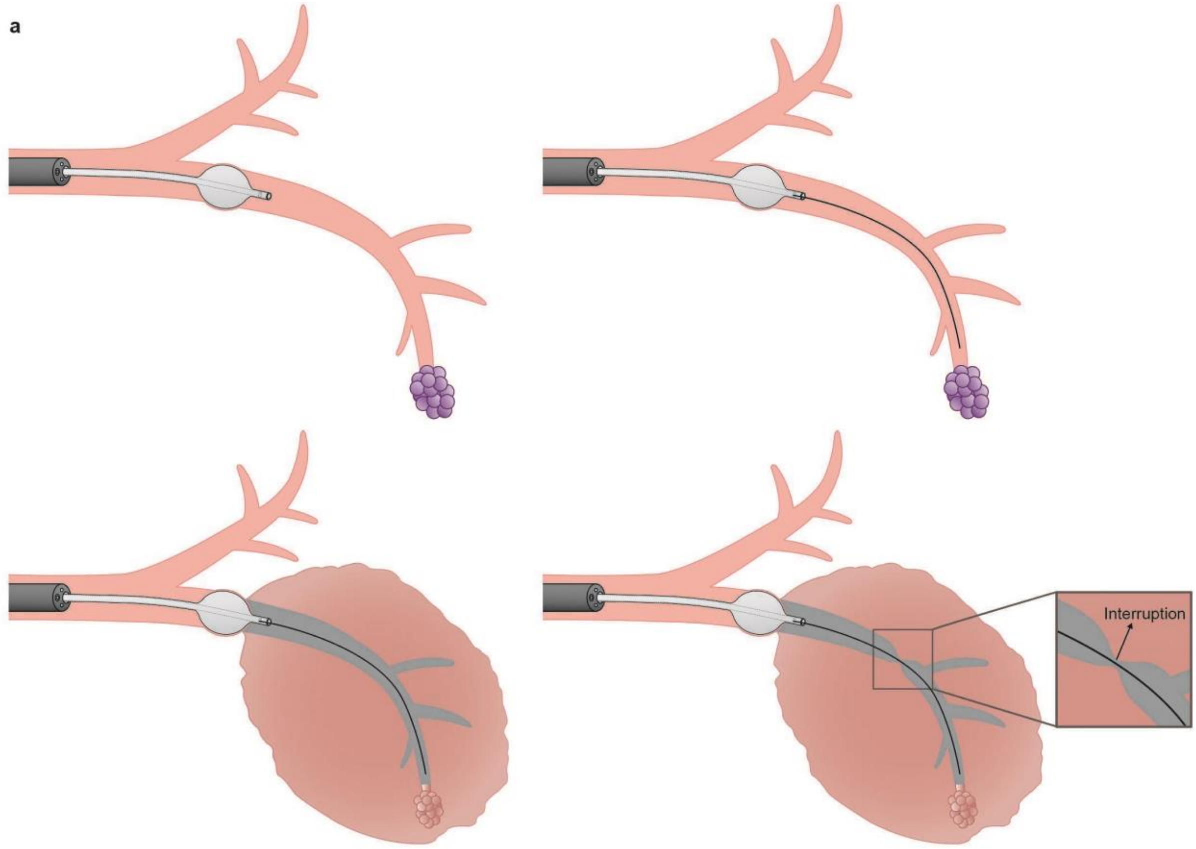

Fig. S3. The use of the guidewire to make up for the possible interruption of the bronchial electrode caused by the local tissue edema during the CAROL ablation.

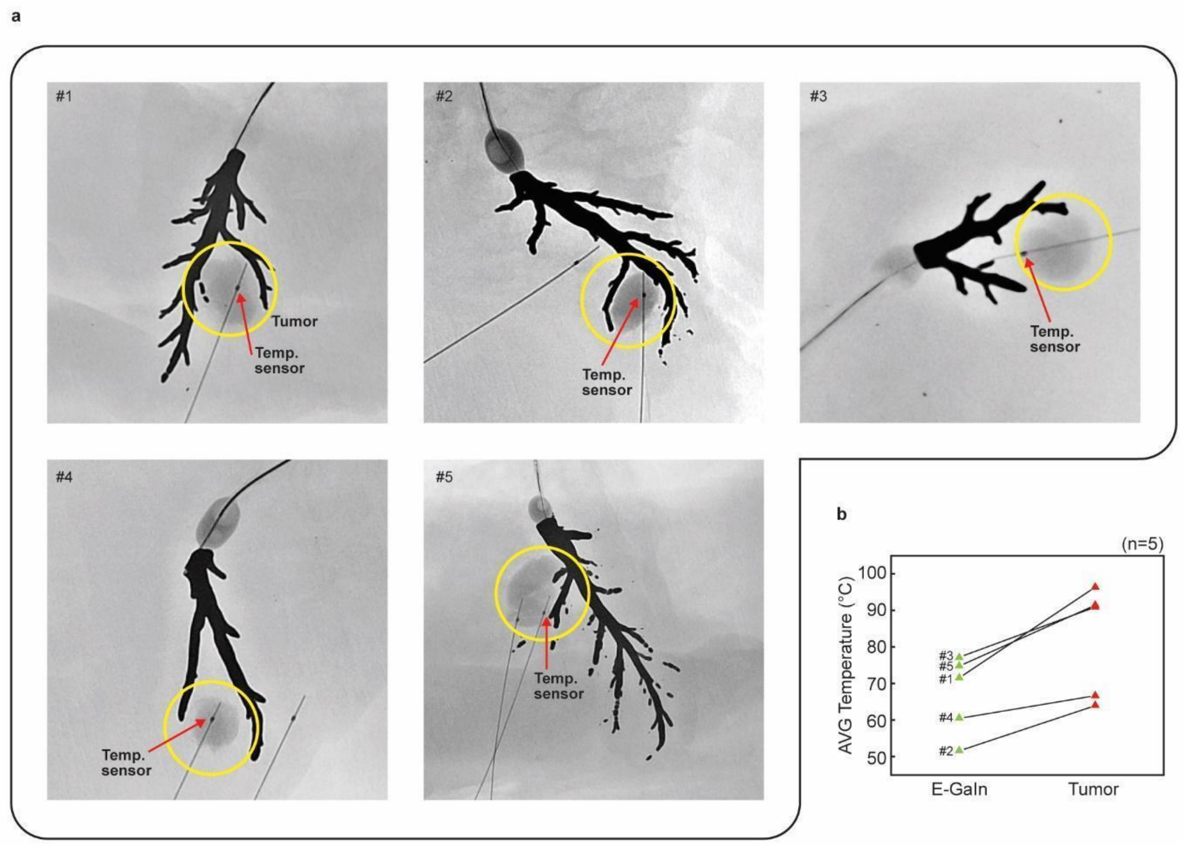

Fig. S4. Experimental results of the Pseudo-tumor ex vivo model. (**a**) Fluoroscopy imaging of Pseudo-tumor model experiments; yellow circle indicates a pseudo-tumor, while red arrow indicates the temperature sensor. (**b**) The average temperature of the pseudo-tumors was higher than that of the central bronchial electrode (p=0.01).

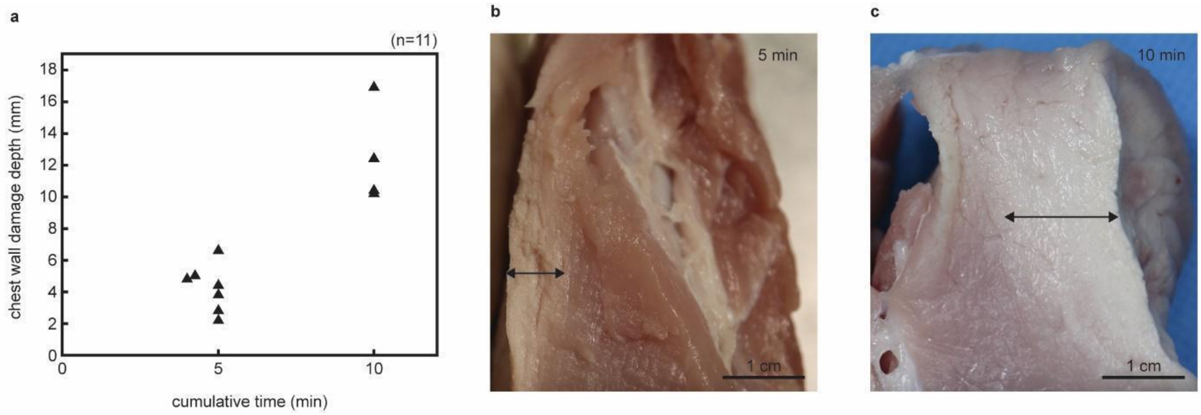

Fig. S5. Model of chest wall damage in ex vivo experiments. (**a**) Depth of Damage in sirloin covering the lungs according to ablation time (4.2 mm ± 1.5 mm at 5 min, 12.5 ± 3.1 mm at 10 min, p=0.01). (**b**) Depth of ablation in the porcine sirloin muscle flap that surrounded the ablated lung segment during CAROL ablation for 5 min. (**c**) Depth of ablated injury after 10 min of CAROL ablation.

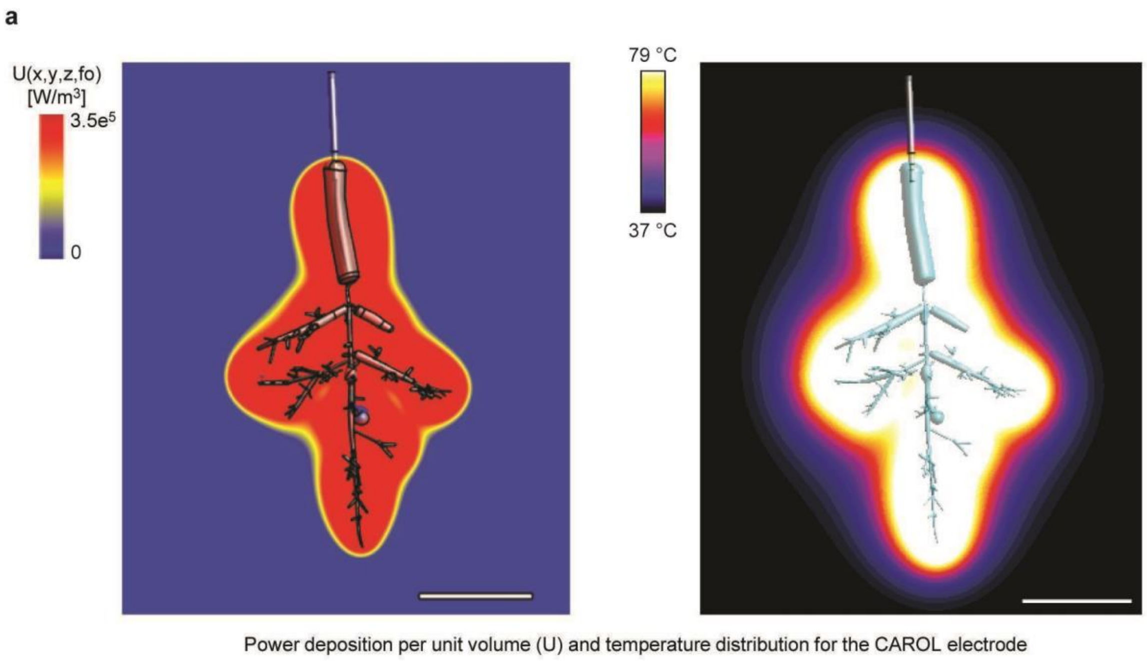

Fig. S6. Based on in-vivo pig experiments of CAROL ablation, data on the thermal conductivity and temperature of the bronchial electrode were obtained through computer simulations.
