# Supplementary material for: Conversion of the bronchial tree into a conforming electrode to ablate the lung nodule in a porcine model
Source: Commun Med (Lond). 2023 Sep 29;3:129. doi: 10.1038/s43856-023-00362-1 (PMC10541426; doi:10.1038/s43856-023-00362-1)
Supplement: Supplementary file 3 — Description of Additional Supplementary Files [file 43856_2023_362_MOESM3_ESM.pdf]

Description of Additional Supplementary Files

File Name: Supplementary Data 1

Description:

Table 1. Total in-vivo table

Table 2. GGO vs hypo area

Table 3. In-vivo blood test

Table 4. Blood concentration

Table 5. E-GaIn suction test

Table 6. Total ex-vivo test

Table 7. Tissue temperature

Table 8. Tumor temperature

Table 9. Depth test

File Name: Supplementary Data 2

Description: In this study, data are summarized as mean ± SD. In the correlation analysis and its test, Pearson's correlation coefficient was used. When comparing, paired t-test was used for paired data, and otherwise, Welch's t-test was used. All statistical analyses were conducted using R (R Statistical Software for Windows, version 4.2.1, 2022, Foundation for Statistical Computing). Statistical significance was set at $P < 0.05$.
