# Supplementary material for: Conversion of the bronchial tree into a conforming electrode to ablate the lung nodule in a porcine model
Source: Commun Med (Lond). 2023 Sep 29;3:129. doi: 10.1038/s43856-023-00362-1 (PMC10541426; doi:10.1038/s43856-023-00362-1)
Supplement: Supplementary file 6 — Reporting Summary [file 43856_2023_362_MOESM6_ESM.pdf]

nature portfolio

Corresponding author(s): Izaz Ali Shah, Hee Yun Seol, Youngdae Cho, Wonjun Ji, Jaeyoung Seo, Cheolmin Lee, Min-Ku Chon, Donghoon Shin, Justin H. Kim, Ki-Seok Choo, Junhui Park, Juhyung Kim, Hyoungsuk Yoo*, June-Hong Kim*

Last updated by author(s): Sep 15, 2023

Reporting Summary

Nature Portfolio wishes to improve the reproducibility of the work that we publish. This form provides structure for consistency and transparency in reporting. For further information on Nature Portfolio policies, see our Editorial Policies and the Editorial Policy Checklist.

Statistics

For all statistical analyses, confirm that the following items are present in the figure legend, table legend, main text, or Methods section.

n/a	Confirmed	
☐	☒	The exact sample size (*n*) for each experimental group/condition, given as a discrete number and unit of measurement
☐	☒	A statement on whether measurements were taken from distinct samples or whether the same sample was measured repeatedly
☐	☒	The statistical test(s) used AND whether they are one- or two-sided<br>*Only common tests should be described solely by name; describe more complex techniques in the Methods section.*
☐	☒	A description of all covariates tested
☐	☒	A description of any assumptions or corrections, such as tests of normality and adjustment for multiple comparisons
☐	☒	A full description of the statistical parameters including central tendency (e.g. means) or other basic estimates (e.g. regression coefficient) AND variation (e.g. standard deviation) or associated estimates of uncertainty (e.g. confidence intervals)
☐	☒	For null hypothesis testing, the test statistic (e.g. *F*, *t*, *r*) with confidence intervals, effect sizes, degrees of freedom and *P* value noted<br>*Give P values as exact values whenever suitable.*
☐	☒	For Bayesian analysis, information on the choice of priors and Markov chain Monte Carlo settings
☐	☒	For hierarchical and complex designs, identification of the appropriate level for tests and full reporting of outcomes
☐	☒	Estimates of effect sizes (e.g. Cohen's *d*, Pearson's *r*), indicating how they were calculated

Our web collection on statistics for biologists contains articles on many of the points above.

Software and code

Policy information about availability of computer code

Data collection	No software was used.
Data analysis	The Analysis codes required to reproduce the results are available Figshare, accessible at the following identifier https://doi.org/10.6084/m9.figshare.23542374.v1<br>In this study, data are summarized as mean ± SD. In the correlation analysis and its test, Pearson's correlation coefficient was used. When comparing, paired t-test was used for paired data, and otherwise, Welch's t-test was used. All statistical analyses were conducted using R (R Statistical Software for Windows, version 4.2.1, 2022, Foundation for Statistical Computing). Statistical significance was set at P < 0.05.

For manuscripts utilizing custom algorithms or software that are central to the research but not yet described in published literature, software must be made available to editors and reviewers. We strongly encourage code deposition in a community repository (e.g. GitHub). See the Nature Portfolio guidelines for submitting code & software for further information.

Data

Policy information about availability of data

All manuscripts must include a data availability statement. This statement should provide the following information, where applicable:

- Accession codes, unique identifiers, or web links for publicly available datasets
- A description of any restrictions on data availability
- For clinical datasets or third party data, please ensure that the statement adheres to our policy

Can download the statistical analysis code by visiting this URL https://doi.org/10.6084/m9.figshare.23542374.v1

Human research participants

Policy information about studies involving human research participants and Sex and Gender in Research.

Reporting on sex and gender	NA
Population characteristics	NA
Recruitment	NA
Ethics oversight	NA

Note that full information on the approval of the study protocol must also be provided in the manuscript.

Field-specific reporting

Please select the one below that is the best fit for your research. If you are not sure, read the appropriate sections before making your selection.

☒ Life sciences ☐ Behavioural & social sciences ☐ Ecological, evolutionary & environmental sciences

For a reference copy of the document with all sections, see nature.com/documents/nr-reporting-summary-flat.pdf

Life sciences study design

All studies must disclose on these points even when the disclosure is negative.

Sample size	This study is pilot study using animal for proof of the concept. So sample size is not applicable to this study
Data exclusions	All experimental data were included in this manuscript.
Replication	We confirmed the reproducibility of our CAROL ablation. It was already expressed in our study methods and results.
Randomization	The experiments were done non-randomly because the data results have nothing to do with randomization.
Blinding	We didn't blinding because the main outcome (tissue ablation) is mainly determined and assessed by imaging (CT) or pathologic evaluation. And all parameters of ablations such as temperature or resistance are something that are very clear, which are not affected by blinding.

Reporting for specific materials, systems and methods

We require information from authors about some types of materials, experimental systems and methods used in many studies. Here, indicate whether each material, system or method listed is relevant to your study. If you are not sure if a list item applies to your research, read the appropriate section before selecting a response.

Materials & experimental systems

n/a	Involved in the study
☒	☐ Antibodies
☒	☐ Eukaryotic cell lines
☒	☐ Palaeontology and archaeology
☐	☒ Animals and other organisms
☒	☐ Clinical data
☒	☐ Dual use research of concern

Methods

n/a	Involved in the study
☒	☐ ChIP-seq
☒	☐ Flow cytometry
☒	☐ MRI-based neuroimaging

Animals and other research organisms

Policy information about studies involving animals; ARRIVE guidelines recommended for reporting animal research, and Sex and Gender in Research

Laboratory animals	Yorkshire farm swine
Wild animals	Yorkshire farm swine were used for the experiment and were properly disposed of in accordance with the regulations of the Korean Food and Drug Administration.
Reporting on sex	There is no difference in effectiveness just because of gender.
Field-collected samples	All experiments were conducted in the laboratory and were performed in compliance with regulations.
Ethics oversight	All experiments were conducted in accordance with ethical approval or guidelines.

Note that full information on the approval of the study protocol must also be provided in the manuscript.
